# Nanotechnological advances in cancer: therapy a comprehensive review of carbon nanotube applications

**DOI:** 10.3389/fbioe.2024.1351787

**Published:** 2024-03-06

**Authors:** Siyang Gao, Binhan Xu, Jianwei Sun, Zhihui Zhang

**Affiliations:** ^1^ Jilin University of College of Biological and Agricultural Engineering, Changchun, Jilin, China; ^2^ School of Mechatronic Engineering, Chang Chun University of Technology, Changchun, Jilin, China

**Keywords:** carbon nanotubes, cancer diagnostics, targeted therapy, biotoxicity, bio-nano interactions

## Abstract

Nanotechnology is revolutionising different areas from manufacturing to therapeutics in the health field. Carbon nanotubes (CNTs), a promising drug candidate in nanomedicine, have attracted attention due to their excellent and unique mechanical, electronic, and physicochemical properties. This emerging nanomaterial has attracted a wide range of scientific interest in the last decade. Carbon nanotubes have many potential applications in cancer therapy, such as imaging, drug delivery, and combination therapy. Carbon nanotubes can be used as carriers for drug delivery systems by carrying anticancer drugs and enabling targeted release to improve therapeutic efficacy and reduce adverse effects on healthy tissues. In addition, carbon nanotubes can be combined with other therapeutic approaches, such as photothermal and photodynamic therapies, to work synergistically to destroy cancer cells. Carbon nanotubes have great potential as promising nanomaterials in the field of nanomedicine, offering new opportunities and properties for future cancer treatments. In this paper, the main focus is on the application of carbon nanotubes in cancer diagnostics, targeted therapies, and toxicity evaluation of carbon nanotubes at the biological level to ensure the safety and real-life and clinical applications of carbon nanotubes.

## Graphical Abstract

**Figure:**
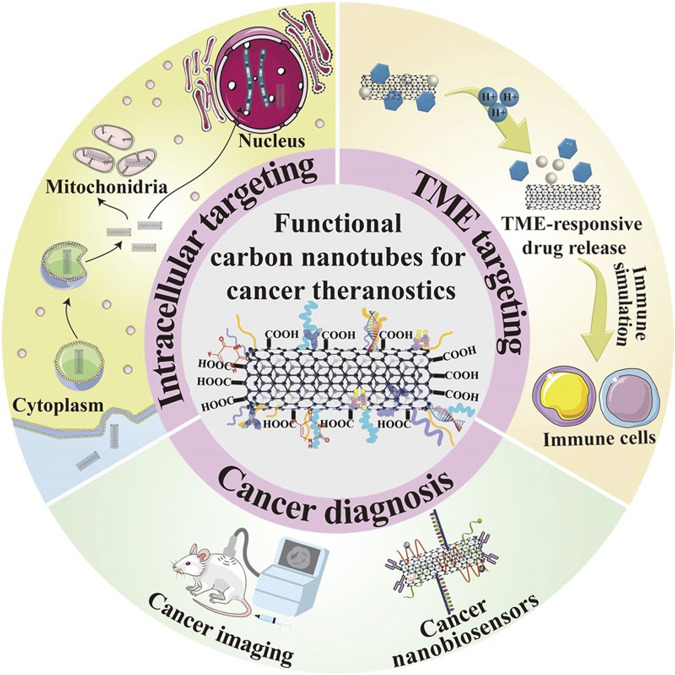
Functional carbon nanotube cancer diagnostics and therapeutics.

## 1 Introduction

**FIGURE 1:**
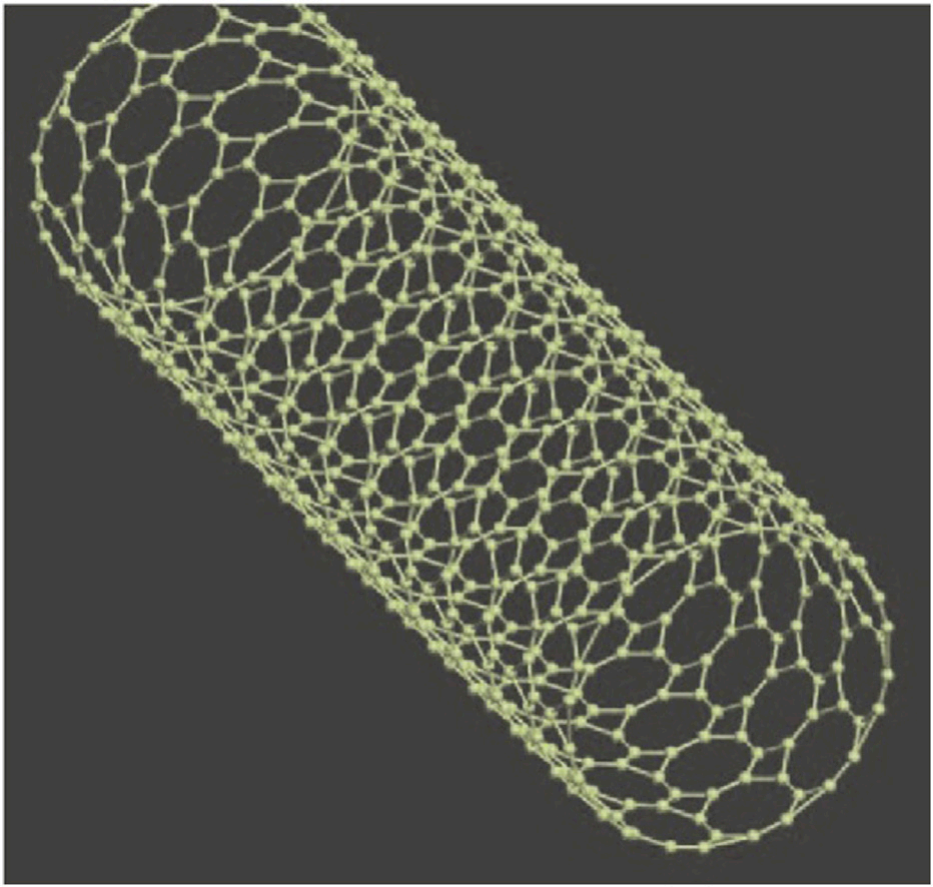
Conceptual diagram of single-walled carbon nanotubes (SWCNT).

**FIGURE 2:**
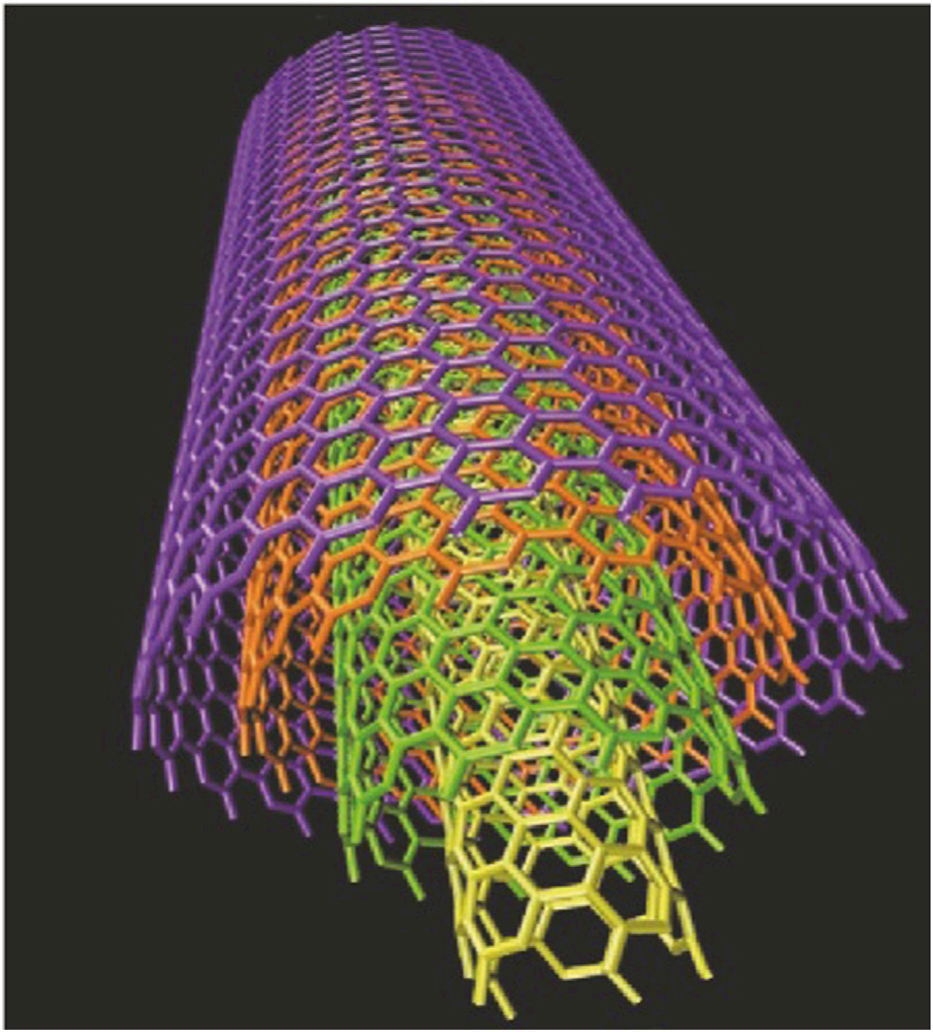
Conceptual diagram of multi-walled carbon nanotubes (MWCNT).

The carbon fiber structure was discovered by Oberlin in 1976 (Takakura et al., 2019) and has attracted much scientific and technological attention around the world due to its potential to revolutionize all areas of nanotechnology, Iijima Sumio discovered carbon nanotubes in 1991, the main techniques used to produce carbon nanotubes include arc discharge (Naik et al., 2021), laser ablation, and thermal and plasma chemical vapor deposition (CVD) (Cai et al., 2021). Carbon nanotubes are classified as single-walled carbon nanotubes (SWCNT) and multi-walled carbon nanotubes (MWCNT). SWCNTs are described as rolled nanotubes, and multi-walled carbon nanotubes contain additional carbon nanotubes around the core. Carbon nanotubes (He et al., 2020), as shown in Figure 1. Single-walled carbon nanotubes (see Figure 2). These tubular structures range from nanometres to tens of nanometres in diameter and up to several centimeters in length, and are usually covered with a fullerene-like structure at their tubular structure. It is believed that the rivet-like properties associated with carbon nanotubes will open new horizons in materials science, especially in the field of conducting polymers and composites (Song et al., 2020). Theoretical and experimental analyses have highlighted that carbon nanotubes have an extremely high modulus of elasticity of >1 TPa, which is comparable to the modulus of elasticity of diamond of 1.2 TPa (Zhang et al., 2019a). The strength per unit weight is reported to be a staggering 10 to 100 times higher than that of the strongest steel (Norizan et al., 2020), further highlighting their superior mechanical integrity. In addition, carbon nanotubes have excellent thermal and electrical properties. It exhibits thermal stability up to 2,800°C in a vacuum, an electrical conductivity of about 103 S/m (Nurazzi et al., 2023), and a thermal conductivity of about 1,900 W/m. (m-K), which is almost twice that of a diamond. Because of these remarkable properties, carbon nanotubes hold great promise for countless scientific and technological applications.

The physical and catalytic properties of carbon nanotubes make them ideal for chemical sensor applications (Norizan et al., 2020). Importantly, the high organization level and high aspect ratio of carbon nanotubes contribute to their excellent chemical, thermal, mechanical properties, and electrical conductivity (Zhang et al., 2020). These unique properties have facilitated the construction of nanoscale devices and the development of multifunctional composites, representing the pinnacle of technological advancement SWCNTs contain sp hybridized carbon and consist of a hexagonal honeycomb lattice-shaped into a hollow tubular structure (Afroze et al., 2021). In contrast, multi-walled carbon nanotubes consist of multiple inter-nested concentric tubes. The diameters of single-walled carbon nanotubes range from 0.4 to 2 nm, whereas the diameters of multi-walled carbon nanotubes range from 1.4 to 100 nm (Panigrahi and Nayak, 2020). Chemical functionalization has emerged as a key approach to enhance dispersibility and reduce toxicity. Functionalized carbon nanotubes (f-CNTs) are made by surface modification of carbon nanotubes to target ligands and drug molecules, thereby reducing toxicity and immunogenicity (Mohanta et al., 2019). These can be used for high drug loading and specific drug delivery to biological systems with significant advantages (Wulf and Bisker, 2022). Due to their unique physicochemical properties, carbon nanotubes have a wide range of applications and drug delivery in various scientific fields such as composites, nanoelectronics (Tang et al., 2021), biosensing, bioimaging, etc. Their relatively lightweight and large surface area make them ideal for the development of drug carriers, components for targeting cellular or subcellular components, and contrast agents. Carbon nanotubes are promising tools for therapeutic and diagnostic applications due to their unique physical and chemical properties and novel tubular structure. These applications have been rigorously studied around the world and significant progress has been made to date. In addition to their established roles in drug delivery and bioimaging (Murjani et al., 2022), carbon nanotubes have shown great potential in cancer therapy. Recent contributions to therapeutic interventions in different types of cancer have highlighted their importance in the field of medicine.

In addition to its role in the diagnosis and treatment of cancer, like any new technology, there are concerns about the application of nanomedicine that stem from a lack of appropriate knowledge and the need to establish regulatory measures. We also need to be aware of its toxic side effects as it travels through the body via the bloodstream. Whether it may cause unnecessary damage to body organs (e.g., liver, kidneys, etc.) is a research question that also deserves our attention.

## 2 The role of carbon nanotubes in cancer diagnosis

Due to their unique electronic, mechanical, and thermal properties, carbon nanotubes are considered to be a promising tool for detecting the expression of biomolecules typical of the early stages of cancer (Coleman et al., 2006). There are currently three medical imaging techniques associated with carbon nanotubes: photoacoustic imaging (PAI) (Kang et al., 2022), fluorescence imaging (FI) (Zhou and Jokerst, 2020), and Raman imaging (Yu et al., 2021a). In photoacoustic imaging, carbon nanotubes have shown advantages in improving imaging signals as contrast agents with strong light absorption in the near-infrared region (NIR) (Gao et al., 2022). In fluorescence imaging, the unique excitation and emission wavelengths of single-walled carbon nanotubes offer the possibility of multicolor fluorescence imaging (Yaghoubi and Ramazani, 2020). Finally, in Raman imaging, isotopic modification of single-walled carbon nanotubes (Gong et al., 2013) enables multicolor Raman imaging of different cancer cells (Ijaz et al., 2023), providing important information about the chemical composition of cells and tissues. These findings highlight the significant potential and application value of carbon nanotubes in modern medical imaging and early cancer diagnosis. In a recent study, scientists have developed a new approach to cancer diagnosis that combines quantum defect-functionalized nanotube arrays with machine learning techniques. The method, which can be called “Cancer diagnosis based on quantum-deficient carbon nanotube arrays and machine learning,” works by obtaining a “disease fingerprint” of an individual with obvious symptoms from the near-infrared fluorescence emission spectra of the carbon nanotube arrays. Different diseases are detected in serum samples. The accuracy of the method is superior to traditional serum-based biomarker identifications such as Cancer Antigen 125 (CA125), Human Epitope 4 (HE4), and YKL40. In addition, the method is very flexible and can be quickly adapted to detect a wide range of diseases/conditions. With sufficient sensor response data and appropriate patient serum samples, the algorithm can be trained to identify almost any cancer. This opens up possibilities for early diagnosis and treatment, as well as providing new tools for future biomarker discovery efforts.

### 2.1 Diagnostic role of Cnt in pancreatic cancer

Pancreatic cancer (PC) has become a prominent and prevalent malignancy within the gastrointestinal tract, casting a long and dangerous shadow due to its extremely high mortality rate and dismal survival prospects (Ahmadian et al., 2022). Its propensity to invade nearby tissues and catalyze distant metastases to other organs further exacerbates the danger. Given the stubborn resistance to conventional chemotherapy, surgical resection is usually the mandatory option. However, the insidious nature of PC masks its early symptoms, resulting in most patients receiving a grim diagnosis only in the advanced stages of the disease (Wang et al., 2011). This delay in detection, coupled with a high propensity for late metastasis, severely compromises the effectiveness of surgical intervention (Saliev, 2019). Despite the introduction of endoscopic ultrasound and computed tomography for PC detection, there are significant inherent barriers to these techniques. The reliability of CT image recognition and skillful endoscopy is highly dependent on the competence of the clinician, which increases the need for innovative early diagnostic methods for PCs.

**FIGURE 3:**
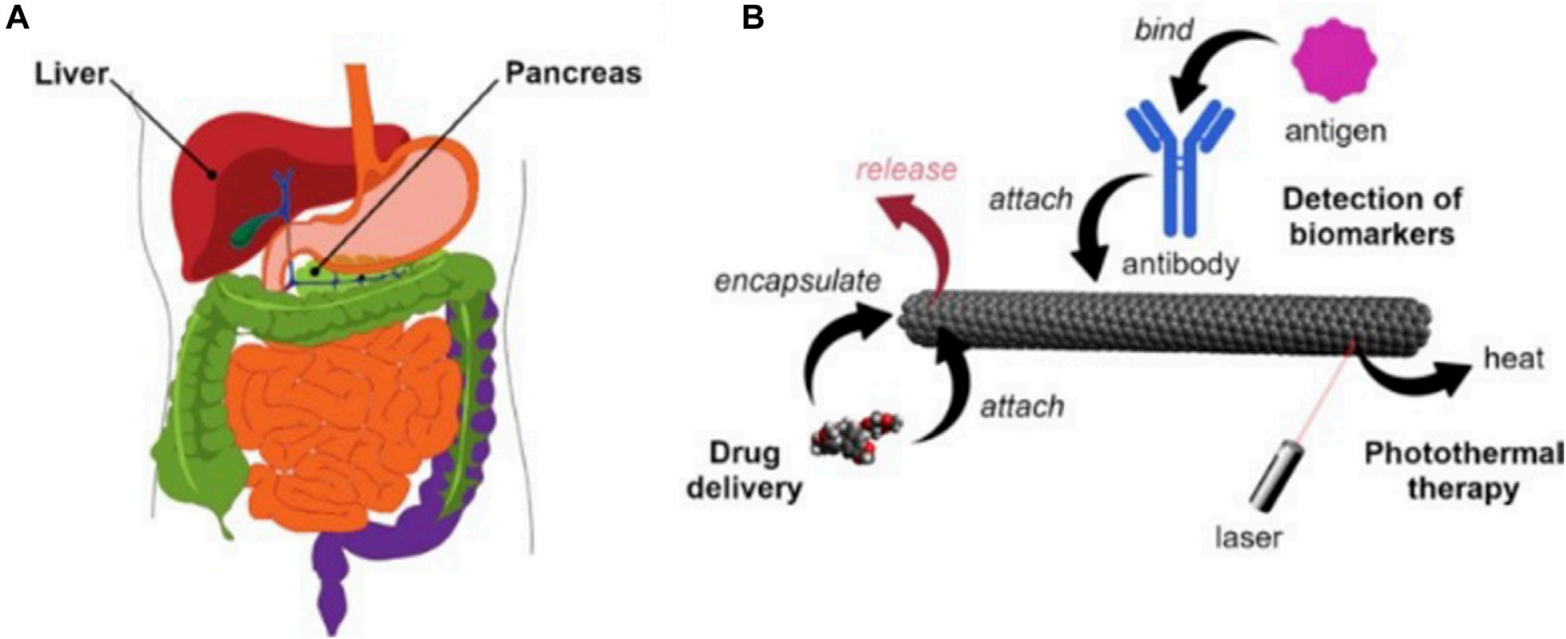
**(A)** Human liver and pancreas **(B)** Main application methods of carbon nanotubes in the diagnosis and treatment of liver and pancreatic cancer.

Three different strategies for the application of carbon nanotubes (Figure 3B). Firstly, carbon nanotubes can be used as templates that can be measured using a variety of optoelectronic methods by mounting a sensing component such as an attached antibody and then altering its electrical or optical properties in response to a specific stimulus (e.g., a cancer protein). Secondly, due to the high absorption of carbon nanotubes in the UV-IR range, appropriate light irradiation can generate heat for the ablation of tumor tissue. Thirdly, carbon nanotubes can be used as nanocarriers loaded with anticancer drugs or functionalized CNTs.Surface modifications are essential to enhance the utility of carbon nanotubes as effective carriers for targeting cancer cells and releasing therapeutic drugs. Previous studies have demonstrated the important impact of these strategies in the treatment of pancreatic and liver cancer (Liu et al., 2021).

### 2.2 Carbon nanotubes in liver cancer diagnosis

Liver cancer is the fifth most common type of cancer in the world, especially in some regions, such as Asia and Africa, where the incidence of liver cancer is relatively high. Traditional treatments include surgical resection, liver transplantation, or radiofrequency ablation. However, these methods are only effective in the early stages. For advanced stages, chemotherapy and targeted delivery are often used. The development of carbon nanotechnology provides a new vehicle for the diagnosis of liver cancer. There are three commonly used markers in the diagnosis of liver cancer, including (α-fetoprotein (AFP), α-fetoprotein variant (AFP- l3), and abnormal plasminogen (APT)) Li and others (Li et al., 2012) constructed au-coated carbon nanotubes conjugated with antibodies labeled with redox probes, which can be used to detect the markers. The principle is that the biomarker attaches to the antibody-immobilized carbon nanotubes to produce a larger signal. In another study, it was found that due to the invasive properties of hepatocellular carcinoma cells highly differentiated cells (HUH7) have a better chance of attachment than poorly differentiated cells (SNU182). Using this property Kucukayan-Dogu et al. (Kucukayan-Dogu et al., 2015) developed an imageable carbon nanotube surface and analyzed the ability of differentiated cells to attach to the imageable carbon nanotube surface. It can be used to diagnose the level of invasion of liver (Figure 3A) cancer cells.

### 2.3 Carbon nanotubes in ovarian cancer diagnosis

Ovarian cancer is the second most common gynecological malignancy worldwide, causing more than 184,000 deaths each year (Ceppi et al., 2019; Markus et al., 2023). When the cancer is detected early and confined to the ovaries, the outlook is relatively positive, with 5-year survival rates exceeding 90 percent. However, when the diagnosis is delayed, the picture takes an ominous turn, with 59 percent of cancer cases breaking through the primary site and metastasizing to distant locations, causing the 5-year survival rate to plummet to 29 percent. This is one scenario. This stark contrast in survival outcomes underscores the critical role of early detection and careful monitoring of disease course and recurrence (Jiang et al., 2023).

**FIGURE 4:**
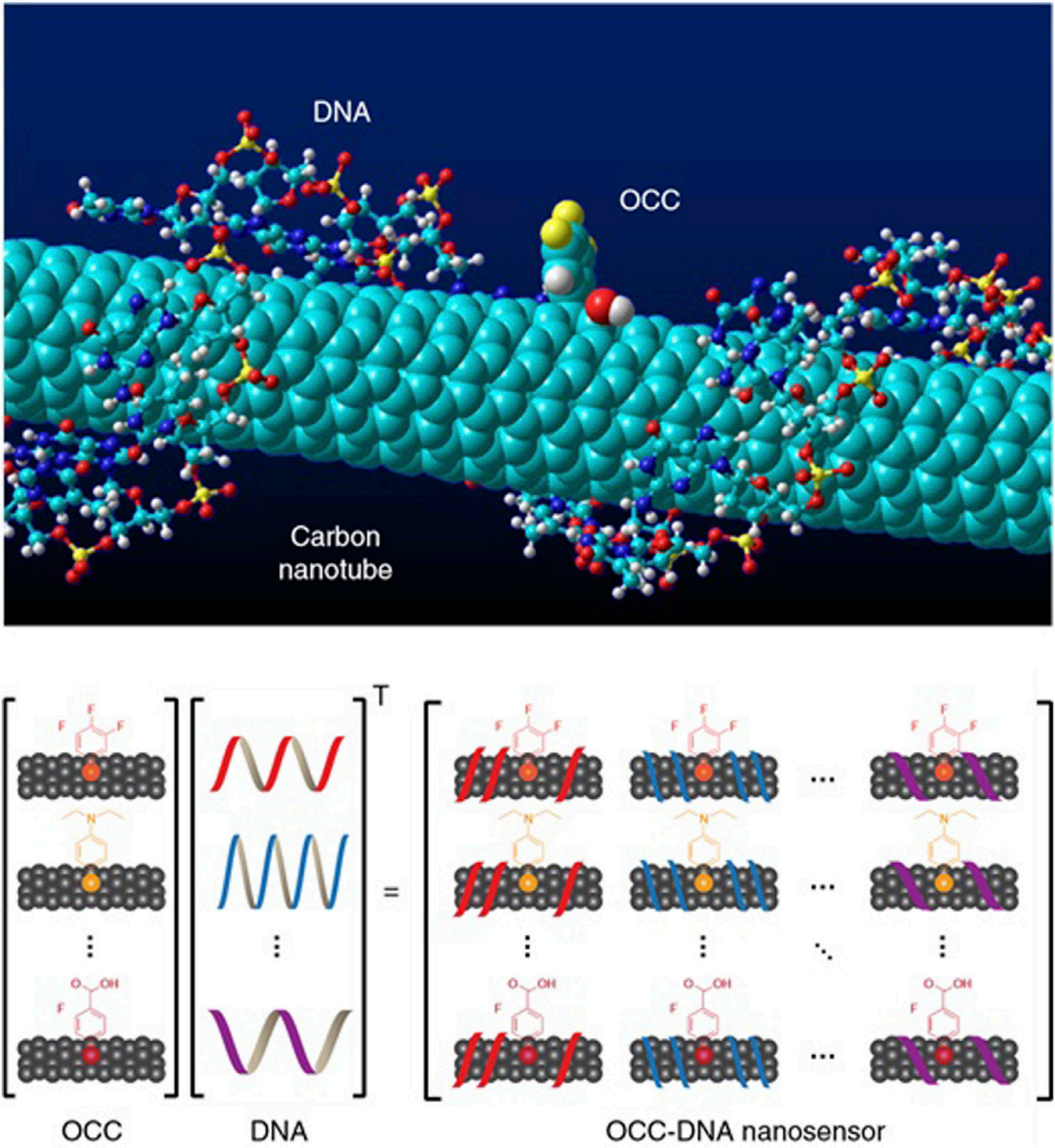
OCC-DNA nanosensor array. Molecular modeling of OCC-DNA nanosensor elements. Shown is (Zhang et al., 2019a; Song et al., 2020)-SWCNT encapsulated by ss(GT)15dnA with 3,4,5 (trifluoroaryl OCC). OCC-DNA nanosensor array constructed from OCC and ssDNA components.

To fill this critical diagnostic gap, Mijin Kim et al. (2022) developed nanosensor arrays. This innovative device encapsulates SWCNT using OCC-functionalized single-stranded DNA (ssDNA), called OCC-DNA, as shown in Figure 4. This design promises promising advances in more timely and accurate detection of ovarian cancer. It will revolutionize the treatment and prognosis of this dreaded disease.

First, they chose as features the spectral variables of the nanotube arrays, which were computed from data on the response of the nanotube arrays to serum samples. They then tested five commonly used machine learning algorithms, including logistic regression (LaValley, 2008), decision trees (Quinlan, 1996), artificial neural networks (Zhang et al., 2019b), stochastic sen (Kumar et al., 2023), and support vector machines, for binary classification with the task of distinguishing patients from patients with other diseases or healthy individuals with ovarian cancer. They then used Bayesian optimization to optimize the hyperparameters of each algorithm (Greenhill et al., 2020). The optimization objective is to minimize (1 - F-score), where F-score is a commonly used metric for classification model evaluation. It is the reconciled mean of precision and recall. In the model training and validation phase, they used a 10-fold cross-validation method (Berrar, 2019), which helps to improve the performance of the model, prevents overfitting, and allows the model to generalize better to new data. In the testing phase, they tested the trained model using an independent test set containing 54 serum samples from different patients. The test results showed that the optimized Support Vector Machine (SVM) model achieved 100% sensitivity and 98% specificity with an F-score of 0.978. These values were consistent with the cross-validation scores, and the Subject Operating Characteristics (ROC) curves were similar, suggesting that the model did not overfit the data. In addition, they constructed a regression model using support vector regression (Huang et al., 2018) (SVR) to predict ovarian cancer serum biomarker levels.

Finally, they tested the trained model using an independent test set containing 54 serum samples from different patients. In addition, they constructed a regression model using support vector regression (SVR) to predict the levels of ovarian cancer serum biomarkers. Carbon nanotubes play a crucial role. Carbon nanotubes are used as highly sensitive optical sensors whose emission is sensitive to the dielectric environment, redox perturbations, and electrostatic charges. By non-covalently encapsulating polymers such as short oligonucleotides, carbon nanotubes can be suspended in water and are molecularly selective. This encapsulation effect defines the shape and size of the exposed surface of the carbon nanotube, allowing the carbon nanotube to produce a specific optical response to analytes in serum. Carbon nanotubes can be used not only as highly sensitive optical sensors but also as a function of machine learning models that enable researchers to diagnose and predict ovarian cancer.

## 3 Carbon nanotubes in cancer therapy

**FIGURE 5:**
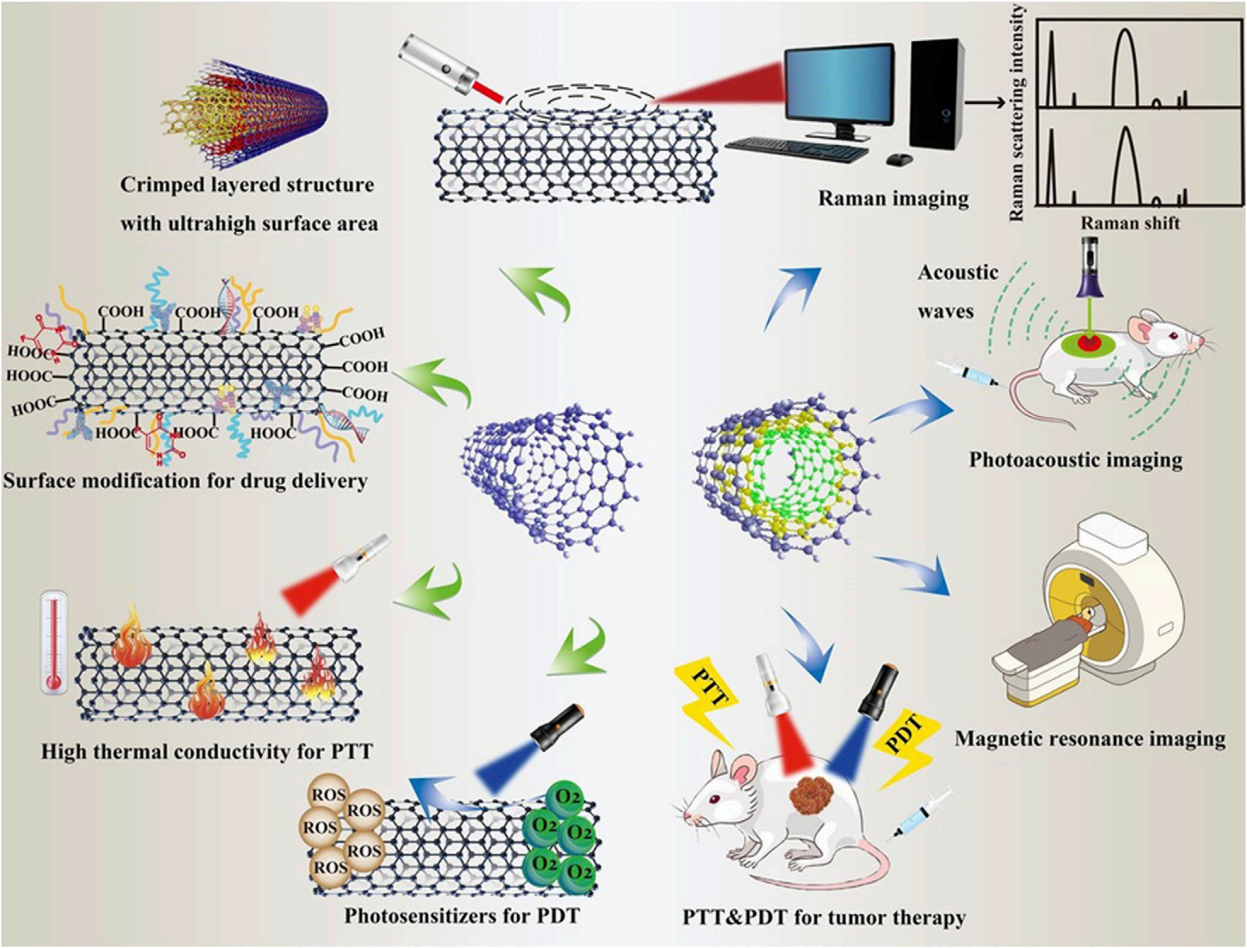
Various applications of CNT in cancer therapy.

Currently, many therapeutic approaches in oncology target tumor cells and their surroundings. Focusing on the tumor cells helps to eradicate the tumor parenchyma directly and effectively. On the contrary, targeting the tumor microenvironment (TME) indirectly eliminates tumor cells by disrupting their basic survival conditions to hinder their proliferation and metastasis. Due to their superior physicochemical properties, drug-carrying surfaces, and ability to penetrate tumors, CNTs are emerging as a new star in the treatment of various cancers in many biomedical fields such as drug delivery, genetic engineering, and biosensing (Figure 5). Therefore, the ensuing discussion will carefully elucidate the mechanisms by which carbon nanotube-based nano-delivery systems can precisely navigate and bind to various therapeutic targets. This includes intracellular sites within tumor cells as well as extracellular components of TME, thus providing a multifaceted approach to cancer therapy.

### 3.1 Nuclear targeting

The cell nucleus is considered an ideal target for malignant tumor therapy because of its role in controlling cell proliferation, metabolism, and cell cycle, as well as activating genes. To treat cancer more effectively, researchers have explored a way to target the nucleus or penetrate the nuclear membrane using nanoparticles. A study conducted by Oh et al. constructed drug delivery systems (Andhari et al., 2020) (DDSs) carrying the topoisomerase inhibitor DOX and polyethylene glycolated single-walled carbon nanotubes (SWNTs) and applied them to near-infrared (NIR)-based photothermal therapy and chemotherapy combination therapy Light irradiation. DOX is a widely used drug for chemotherapy, but its nonspecific distribution and side effects caused by high-dose administration limit its further application. Therefore, the effective delivery of DOX can be improved by utilizing high surface area SWNT as a carrier. The results showed that synthetic PEG-SWNT-DOX could effectively lead to breast cancer cell death compared to DOX or photothermal therapy alone. In addition, carrier-released DOX could accumulate in high concentrations in cancer cells and effectively localize in the nucleus, suggesting that PEG-SWNT-DOX has a promising potential in breast cancer therapy.

**FIGURE 6:**
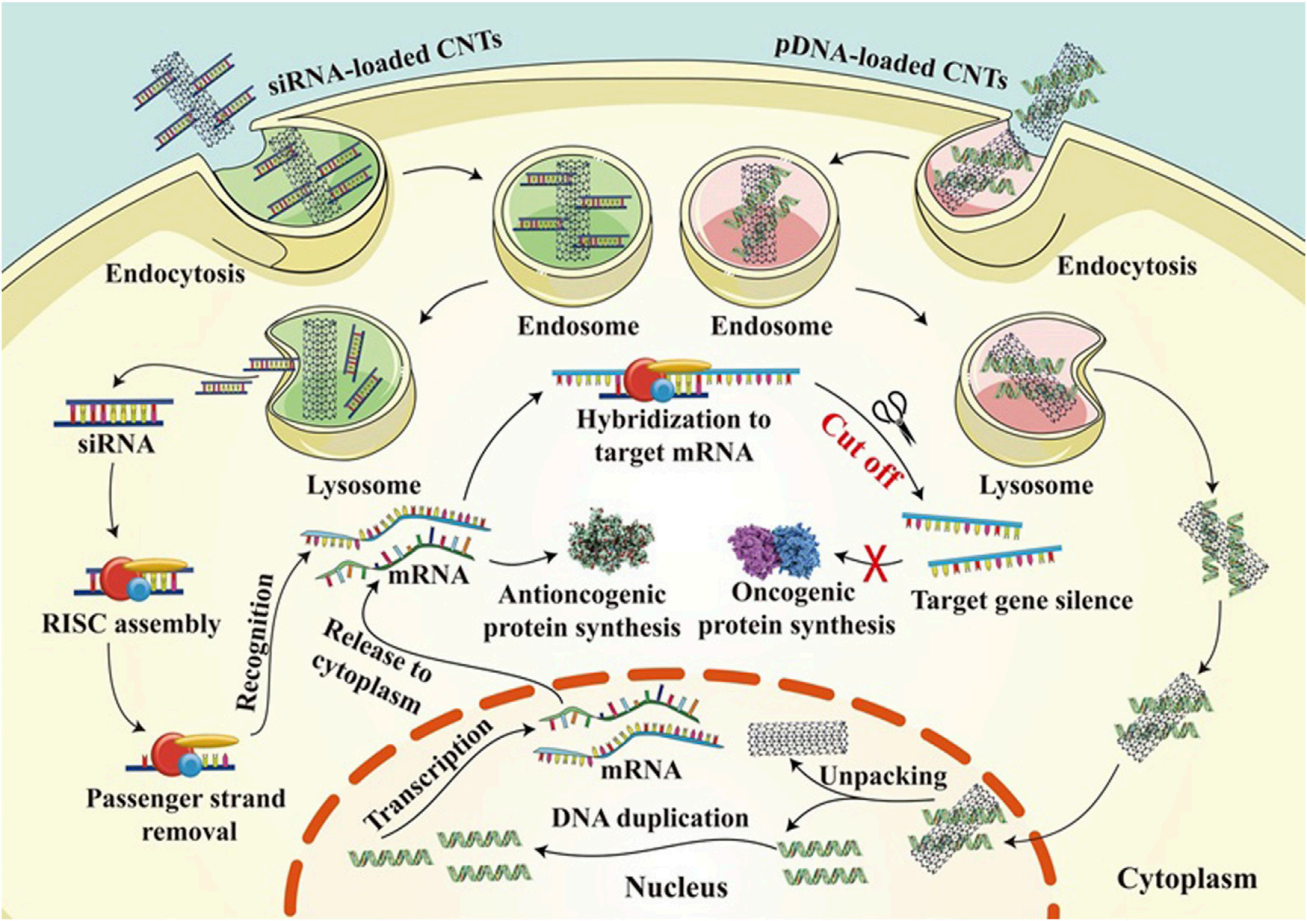
Gene therapy mechanisms of CNT loaded with siRNA and CNT loaded with pdna in cancer treatment.

These studies suggest that nanoparticles as drug delivery systems can improve the effectiveness of cancer therapy by targeting the nucleus. By selecting appropriate carriers and drugs, efficient drug delivery and precise release of drugs can be achieved, thus reducing damage to normal cells during treatment and improving therapeutic efficacy. However, these studies are still in the laboratory stage, and further research and clinical trials are needed to verify their potential for practical application in cancer therapy. Gene therapy is an emerging field aimed at correcting genetic diseases at the molecular level. Gene therapy focuses on the use of plasmid DNA (pDNA) to carry genes to increase the expression of downregulated genes and alter mutated genes, or to interfere with the levels of RNA RNA (shRNA) through the application of small interfering RNAs (siRNAs), microRNAs (miRNAs), and short hairpins to reduce protein expression (Figure 6) (Teo et al., 2016). One of the critical success factors is an effective gene delivery vector, which needs to protect the exogenous DNA from degradation and inactivation while avoiding intracellular lysosomal degradation. In this regard, carbon nanotubes have attracted much attention as non-viral gene carriers. Carbon nanotubes interact with many biological components for efficient internalization in various cell types through different mechanisms. Non-viral vectors usually exhibit better immunogenicity and reduced immunogenic side effects on the organism. Use of functionalized carbon nanotubes as carriers for breast cancer therapy using the oncogene suppressor protein 53 (p53) gene as a model gene (Karmakar et al., 2011). The complex has been shown to enter breast cancer cells and release the p53 gene in the nucleus, which activates the apoptotic pathway in the cytoplasm through transcription and translation, leading to therapeutic effects.

### 3.2 Cytoplasmic targeting

Multi-walled carbon nanotubes (MWNT) co-deliver the multi-targeted kinase inhibitor sorafenib (Sor) and EGFR siRNA for liver cancer treatment (He et al., 2017). Sor is a novel multi-targeted anticancer drug and a small-molecule multikinase inhibitor. Its anticancer effect is mainly through the inhibition of several receptor tyrosine kinases closely related to tumor angiogenesis and cell proliferation, thus destroying the tumor microvessels, and ultimately inhibiting tumor growth. The drug is mainly used to treat patients with solid tumors such as advanced renal cell carcinoma (RCC) and advanced hepatocellular carcinoma (HCC). Sor specifically targets liver cancer and is the first drug proven to improve the condition of patients with hepatocellular carcinoma. EGFR plays a key role in tumor development and progression and has become an important target for cancer treatment. There is a class of anticancer drugs called EGFR inhibitors, which can specifically inhibit the activity of EGFR or prevent the binding of EGFR to its ligands, thus inhibiting the activation of its downstream signaling pathway and achieving the goal of inhibiting tumor growth. EGFR inhibitors have been successfully used in the treatment of a variety of tumors, including non-small cell lung cancer, colorectal cancer, and head and neck cancer.

*In vivo* experimental data in mice further demonstrated that treatment with the nanocomplexes significantly reduced tumor volume and weight in homozygous mice, suggesting its excellent efficacy in the treatment of hepatocellular carcinoma. In addition to the above examples, Kirkpatrick et al. used single-walled carbon nanotubes (SWNT) to deliver hypoxia-inducible factor 1α (HIF-1α) siRNA to pancreatic cancer cells (Kirkpatrick et al., 2012), to inhibit HIF-1α expression. The expression of hypoxia-inducible factors, especially HIF-1α, is abnormally increased in many types of tumors. They promote tumorigenesis and progression by inducing the expression of a series of cell growth-promoting, anti-apoptotic genes. Therefore, tumor hypoxia is considered a potential therapeutic target, and many studies have reported that inhibition of HIF-1α can suppress tumor growth by reducing its activity (Rashid et al., 2021). This nanocomplex can effectively achieve the target of RNAi and effectively inhibit tumor growth, further demonstrating the critical role of CNT as a good siRNA delivery platform (Gao et al., 2021). In addition to SWNT and MWNT, double-walled carbon nanotubes (DWNT) that bridge the gap between SWNT and MWNT can also be used for siRNA delivery. Neves et al. used peptide-coated DWNT loaded with survivin siRNA to treat prostate cancer (Asefifeyzabadi et al., 2021). Due to the nano-needle structure of DWNTs, they can effectively enter mammalian cells and escape the lysosome, releasing siRNA into the cytoplasm, inhibiting the synthesis of survivin proteins, and directly leading to apoptosis of cancer cells. Overall, carbon nanotube-based nano drug delivery systems are promising tools for delivering siRNA into the cytoplasm for effective gene therapy.

### 3.3 Tumor microenvironment-responsive drug release

During cancer progression, tumor heterogeneity increases as the cellular and non-cellular components of the tumor ecological niche, the tumor microenvironment (TME), mature. Tumor initiation is based on a complex series of events that occur in normal cells, and these complex biological events will lead to proliferation, uncontrolled growth, and resistance to cell death. As tumor cells continue to proliferate, the size of the tumor increases with associated TME remodeling. The potential of carbon nanotube-based nanoplatforms for application in cancer therapy, particularly their ability to respond to TME-specific conditions, was demonstrated by Yang et al. The system designed by Yang et al. consisted of oxidized multi-walled carbon nanotubes (MWNTs) with a large inner diameter to encapsulate the anticancer drug cisplatin in its inner structure, and the outer surface was loaded with doxorubicin (DOX) with the addition of polyethylene glycol (PEG) and folic acid, to block the premature release of cisplatin. The cytotoxicity of this nanosystem was more pronounced in an acidic environment at pH 6.5 than at pH 7.4, confirming its potent antitumor effect in acidic TME. Wang et al. modified uniform manganese dioxide and the photosensitizer chloroaluminum phthalocyanine (Ce6) on MWNTs for enhanced MRI-guided and TME-responsive phototherapy. Manganese dioxide was able to catalyze the decomposition of hydrogen peroxide in the tumor microenvironment (TME) to generate single-linear oxygen. This catalytic reaction enhances the photothermal effect produced by multi-walled carbon nanotubes (MWNTs), i.e., the generation of heat by light irradiation, which is used to destroy tumor cells.

### 3.4 Hyperbranched multifunctional carbon nanotube carriers for targeted therapeutics

Mariappan Rajan’s Research focuses on how to overcome the systemic toxicity of anti-cancer drugs, which often limits the use of conventional chemotherapy in cancer treatment (Zhou et al., 2022). To address this issue, researchers have developed a multifunctional nanocarrier system that can precisely deliver anticancer drugs into cancer cells.

**FIGURE 7:**
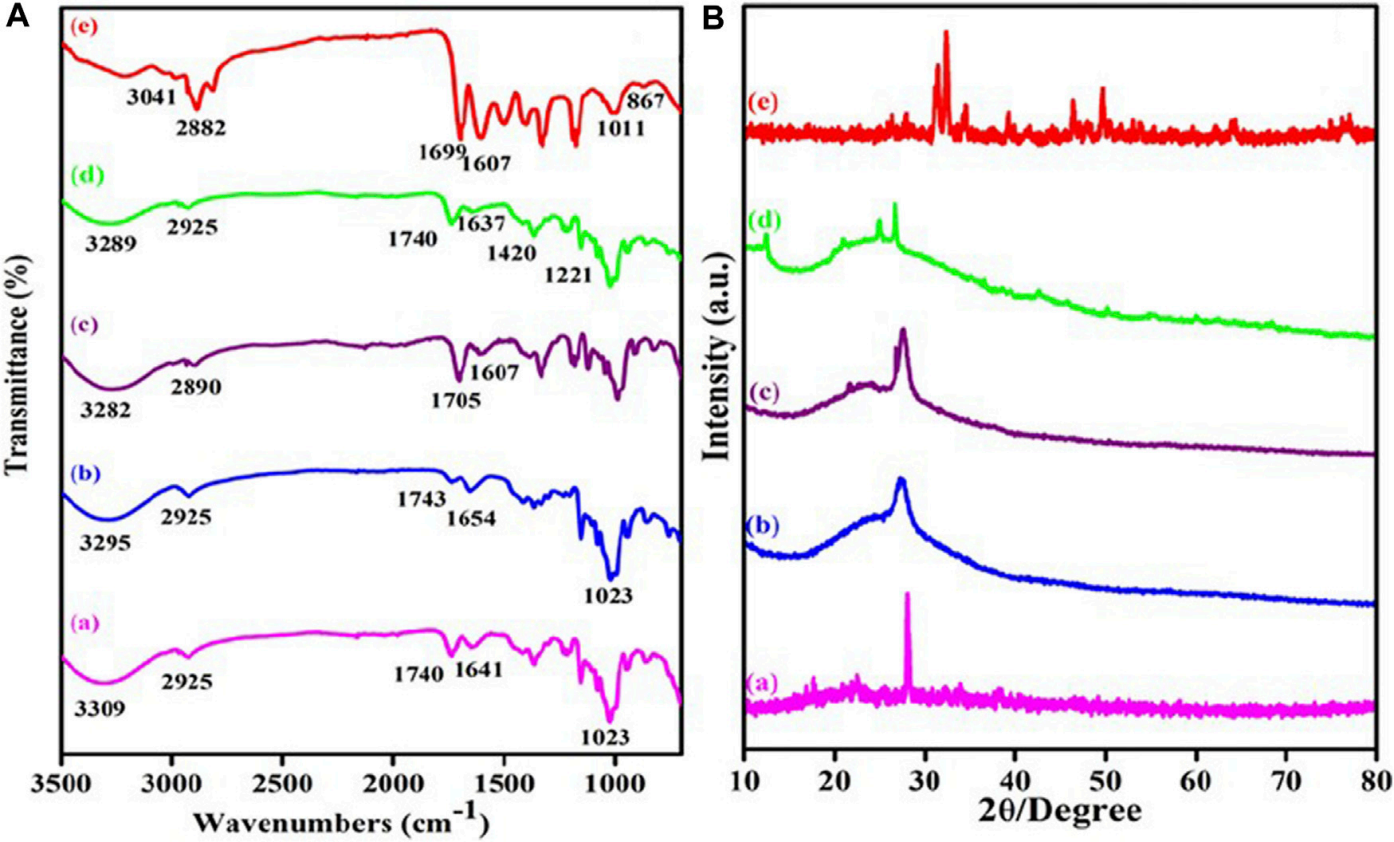
**(A)** FTIR spectra of (a) MWCNT-COOH, (b) MWCNT-PEG, (c) MWCNT-PEG-AA, (d) MWCNT-PEG-AA-HBPLL-FA carrier and (e) DOX/MWCNT-PEG-AA-HBPLL-FA carrier and **(B)** XRD pattern of (a) MWCNT-COOH, (b) MWCNT-PEG, (c) MWCNT-PEG-AA, (d) MWCNT-PEG-AA-HBPLL-FA carrier and (e) DOX/MWCNT-PEG-AA-HBPLL-FA carrier.

The researchers used a variety of techniques to study the chemical functionalization, morphological properties, crystalline properties, surface charge, and thermal stability of the synthesized materials. Through FT-IR spectroscopy, the researchers found structural changes in MWCNT-COOH, MWCNT-PEG, MWCNT-PEG-AA, MWCNT-PEG-AA-HBPLL-FA, and DOX/MWCNT-PEG-AA-HBPLL-FA carriers (Zhou et al., 2022). For example, the FT-IR spectra of MWCNT-PEG showed features of carboxylic acid MWCNT, indicating that the PEG had successfully reacted with the carboxylic acid groups on the surface of MWCNT (Han et al., 2011). In addition, the FT-IR spectra of MWCNT-PEG-AA showed new ester bonds, indicating that the fatty acid had successfully bound to MWCNT-PEG. Finally, the FT-IR spectra of the DOX/MWCNT-PEG-AA-HBPLL-FA carrier showed new amino and carbonyl absorption peaks, indicating that DOX had successfully bound to the carrier (Purkait and Sontakke, 2023). By XRD analysis, the researchers found that the XRD pattern of MWCNT-COOH showed typical 002 crystalline surface diffraction peaks, indicating that MWCNT-COOH has good crystallinity. Upon reaction with PEG, the intensity of the diffraction peaks of MWCNT decreased, indicating that PEG had successfully bound to the MWCNT surface. In addition, the XRD patterns of MWCNT-PEG-AA-HBPLL-FA and DOX/MWCNT-PEG-AA-HBPLL-FA carriers showed new diffraction peaks, suggesting that FA and HBPLL have been successfully bound to MWCNT-PEG-AA. As shown in Figure 7. The researchers were able to reveal structural changes in MWCNT-COOH, MWCNT-PEG, MWCNT-PEG-AA, MWCNT-PEG-AA-HBPLL-FA and DOX/MWCNT-PEG-AA-HBPLL-FA carriers. These techniques can provide detailed information about the chemical and physical properties of the materials, including the presence of chemical bonds and crystal structures (García-López and Palmisano, 2021).

**FIGURE 8:**
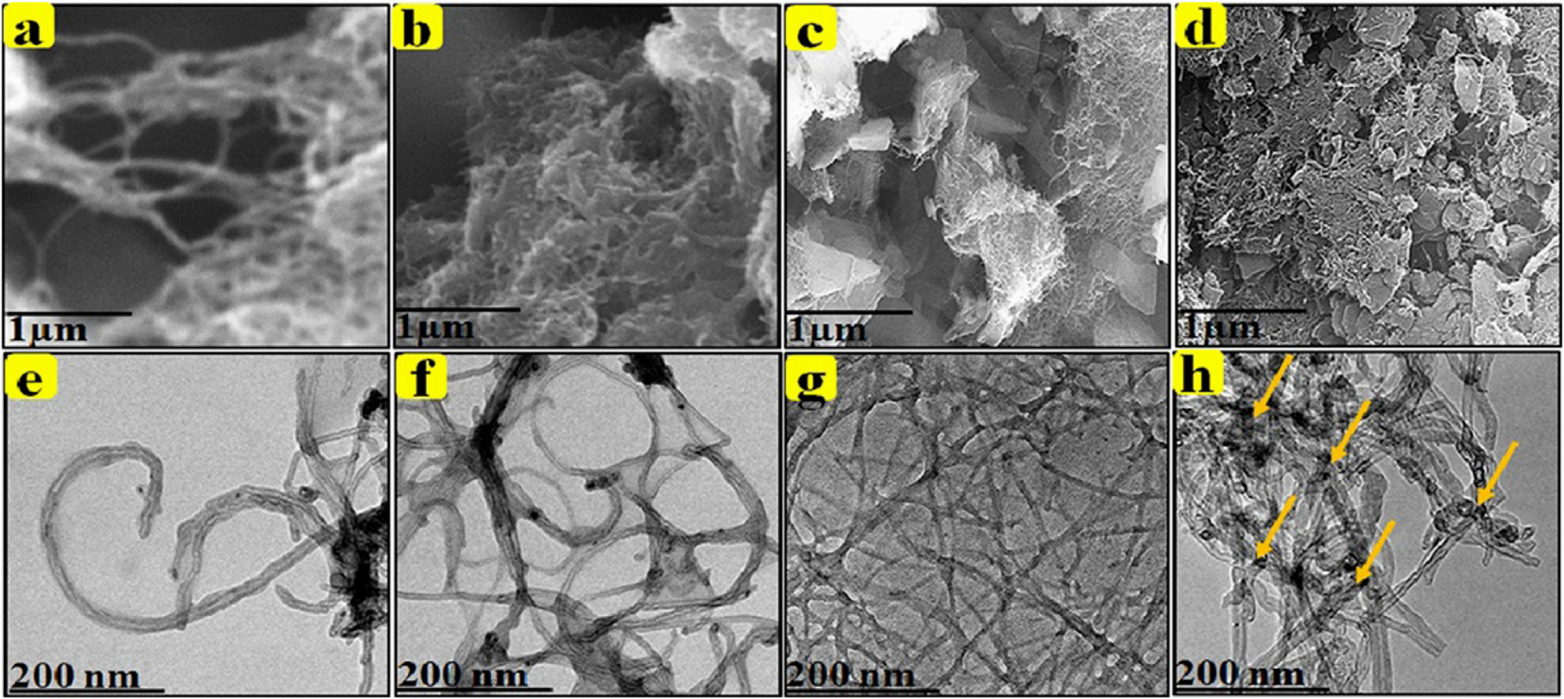
SEM and HR-TEM images of MWCNT-COOH, MWCNT-PEG, MWCNT-PEG-AA-HBPLL-FA, and DOX/MWCNT-PEG-HBPLL-AA-FA carriers. **(A)** MWCNT-COOH clearly shows tubular structure. **(B)** After the reaction of PEG in MWCNT, the edges of MWCNT showed PEG-attached cluster-like structures. **(C)** The curly tubular structure also indicates successful coupling of FA and MWCNT-PEG-AA. **(D)** DOX molecules were loaded onto the surface of MWCNT-PEGHBPLL-AA-FA to obtain sporadic coiled coil-like structures. **(E)** HR-TEM images demonstrated that MWCNT-COOH has a tubular structure. **(F)** shows the presence of hydrophilic PEG on the edge of the grafted MWCNT surface. **(G)** MWCNT-PEG-AA and FA were attached to the polymer material of HBPLL to form an enlarged polymer mesh structure as shown. **(H)** Drug molecules bound to MWCNT-PEG-AAHBPLL-FA carriers showing interconnected mesh structure.

The use of high-resolution transmission electron microscopy (HR-TEM) provides high-resolution images of the intrinsic surface morphology of CNT-based carriers and drug carrier systems. This technique is important for understanding the morphological and structural properties of nanocarrier systems. In an *in vitro* cytotoxicity study (Mohajer et al., 2023), the researchers found that the drug-loaded DOX nanocarrier exhibited increased cytotoxicity and apoptosis in hepatic HepG2 cells (shown in Figure 8). This result suggests that the nanocarrier may be a potential drug carrier for liver cancer chemotherapy. In addition, microscopic analysis using Hoechst 33,342 dye allowed visualization of nuclear damage in the cells. This technique is useful for assessing the toxicity and effects of drugs on cells. *In vitro*, drug release studies using a UV-visible spectrophotometer allow accurate measurement of the rate and total amount of drug released (Ozlu et al., 2019). The release rate of DOX from the carriers reached up to 92.0% within 24 h at three different physiological pH values (2.8, 5.5, 6.8, and 7.4), indicating that the nanocarrier system has good drug release properties.

They investigated the *in vitro* cytotoxicity and anticancer properties of DOX drug-loaded nanocarriers in human hepatocellular carcinoma (HepG2) cells and human embryonic kidney (HEK293) cells (Soma et al., 2000). The results showed that drug-loaded DOX nanocarriers exhibited increased cytotoxicity and apoptosis in hepatic HepG2 cells. The importance of this study is that it provides a possible new approach to improve the targeting of anticancer drugs and reduce their systemic toxicity (Wei et al., 2023). By using a nanocarrier system, the researchers were able to precisely deliver the anti-cancer drug to the cancer cells, thus avoiding the toxicity of the drug to healthy cells. This approach also improves the effectiveness of the drugs because they directly target the cancer cells rather than being dispersed throughout the body. Another important contribution of this research is that it demonstrates how multiple techniques can be used to study and optimize nanocarrier systems. This includes the use of UV-Vis spectrophotometry to study drug release, as well as the use of a variety of techniques to study the chemical functionalization, morphological properties, crystalline properties, surface charge, and thermal stability of synthetic materials. These techniques provide researchers with important information about the performance of nanocarrier systems, helping them to optimize the design and performance of their systems.

Overall, this research opens up new possibilities for cancer treatment. By using nanocarrier systems, it may be possible to deliver anticancer drugs to cancer cells more efficiently, thereby improving treatment efficacy and reducing side effects. However, this research is still in its preliminary stages and further studies are needed to validate the effectiveness and safety of this approach (Witkowska et al., 2022; Rashid et al., 2023).

## 4 Carbon nanotubes: bio interfacial toxicity assessment

Due to the unique structure of carbon nanotubes, they have potential in drug delivery mechanisms, so a lot of work has been put into making them more useful in the pharmaceutical field (Iijima, 1991). After delivery of anticancer drugs to the target cells, CNTs are transported through the bloodstream to organs such as the heart, lungs, liver, kidneys, brain, embryo, etc (Yan et al., 2019) to generate oxidative stress and cause cellular damage. Due to their special surface properties and small size, even purified CNTs can be toxic to tissues or organs. For example, in an experiment by Tsuji et al. in which 100 μg/mL of untreated and purified SWCNTs were co-cultured with HeLa cells for 48 h, apoptosis was observed in 70% of the HeLa cells in the untreated group as compared to 40% of the HeLa cells in the purified group, which supports the theory that CNTs are intrinsically toxic, regardless of the purity of the preparation. Therefore, it is necessary to consider the type of potential toxicity of antitumor nanopreparations prepared from carbon nanotubes on human organs. The relevant toxicities induced by carbon nanotubes include hepatotoxicity, pulmonary toxicity, and cardiovascular toxicity. As the use of carbon nanotubes in improving drug delivery becomes more common, their exposure to humans is bound to accelerate in the future. Therefore, it is important to study issues related to carbon nanotube toxicity.

### 4.1 Cardiovascular toxicity

The interaction of carbon nanotubes with cardiomyocytes is associated with cardiovascular toxicity and potential heart-related risks. These include abnormal cell proliferation, muscle tissue damage, blood flow disturbances, and the development of atherosclerosis. These findings emphasize the importance of assessing the cardiovascular effects of carbon nanotube exposure (Ceriello and Motz, 2004). Many experimental studies have emphasized oxidative stress and inflammation as the main causes of cardiovascular toxicity. Various experiments hypothesized that carbon nanotubes have toxic effects on cardiomyocytes (Garibaldi et al., 2005).

In this *in vitro* study, cells of the rat cardiac cell line H9c2 were isolated and then exposed to high-purity single-walled carbon nanotubes (Ning et al., 2007) (SWCNT) at a concentration of 0.2 mg/mL. The device was designed to evaluate the effects of single-walled carbon nanotubes on cardiomyocytes under controlled laboratory conditions (Hosseinpour et al., 2016). Under microscopic observation, the carbon nanotubes were found to adhere to the cell membrane of H9c2 cardiomyocytes, causing subtle morphological changes. The study concluded that prolonged exposure to carbon nanotubes may lead to severe cardiovascular toxicity, emphasizing the potential risk of these nanomaterials to heart health (Shah et al., 2021).

In another study, hypertensive rats were given soluble single-walled carbon nanotubes, and physiological measurements were performed to assess toxic effects. Notably, malondialdehyde levels changed significantly, suggesting that the carbon nanotubes caused potential oxidative stress. In addition, these nanotubes were found to affect reactive nitrogen levels and alter the activity of nitric oxide synthase (a key enzyme in nitric oxide production), suggesting a complex interaction with cardiovascular health factors.

The narrative surrounding multi-walled carbon nanotubes (MWCNTs) similarly involves cardiovascular toxicity. In a well-known study, multi-walled carbon nanotubes of different lengths were introduced into two different groups of rats (spontaneously hypertensive rats and Wistar-KYOTO rats) via endotracheal injection. This study aimed to evaluate the cardiovascular effects of multi-walled carbon nanotubes in different physiological environments (Ibrahim et al., 2006). The study showed that spontaneously hypertensive rats showed a sustained decrease in heart rate and lower blood pressure after exposure to multi-walled carbon nanotubes. This result mirrors that observed in experiments involving single-walled carbon nanotubes, suggesting a consistent pattern of cardiovascular effects associated with carbon nanotube exposure.

These experimental observations focus on a common premise: carbon nanotube deposition within myocardial vascular tissue leads to increased levels of oxidative stress and inflammation (Joviano-Santos et al., 2014). In general, carbon nanotubes (Thostenson et al., 2001), both single-walled and multi-walled, exhibit some toxic effects on the cardiovascular system, especially in animal models of hypertension (Yu X.-J. et al., 2021b). These toxic manifestations are mainly due to amplification of oxidative stress and inflammation, as well as disruption of biochemical pathways important for cardiovascular function. This series of findings emphasize the need for a thorough evaluation and careful approach to the biomedical applications of carbon nanotubes, especially in cardiovascular health.

### 4.2 Cytotoxicity

Carbon nanotubes are nanomaterials with potential biomedical applications, but their toxicity to cells is also an important aspect of research. Different carbon nanotube structures and surface modifications may affect their biodistribution and cytotoxicity. The pathway of carbon nanotubes into cells through the lipid bilayer of the cell wall (Heister et al., 2013) has been repeatedly explored. Several studies have shown that endocytosis is the mechanism by which carbon nanotubes enter cells (Witzmann and Monteiro-Riviere, 2017). The passage of carbon nanotubes through the lipid bilayer of cells induces oxidative stress, which may lead to inflammation and cause cytotoxicity. Since carbon nanotubes are foreign to the cell, they trigger a foreign body reaction that releases chemicals to expel the nanotubes from the cell. Carbon nanotubes have been observed to induce cellular oxidative stress. Protein kinase and nuclear factor Kappa B are major signaling factors (Wani et al., 2011) that regulate cytokine responses to carbon nanotube-induced oxidative stress. Several experiments have shown that organs such as the spleen, kidneys, and lungs are susceptible to oxidative stress induced by free radical formation. Another mechanism of toxicity is the production of reactive oxides (ROS). ROS are usually by-products of oxygen metabolism processes. This is because carbon nanotubes increase oxidative stress within the cell, thereby increasing ROS levels. Elevated levels of reactive oxygen species (ROS) can have deleterious effects on cell structure and function, including triggering apoptosis, causing damage to genetic material, oxidizing amino acids, and inactivating enzymes. In a pivotal study by Wang et al. (2011), PC12 cells were placed in an environment containing single-walled carbon nanotubes at a concentration of 200 mg/mL (Fogden et al., 2012). This exposure resulted in significantly elevated levels of ROS in the treated cells, which increased approximately five-fold compared to baseline in untreated cells. In addition, the inflammatory response induced by carbon nanotube exposure correlated with the onset of toxicity within the biological system. The study emphasized “impaired phagocytosis” as a key factor in the inflammatory response, i.e., the inability of macrophages to phagocytose carbon nanotubes. Poland et al. showed that while exposure of mice to carbon black induced a normal immune response in which foreign bodies were recognized and eliminated, multi-walled carbon nanotubes and asbestos led to a different response (Poland et al., 2008). Macrophages are unable to destroy these substances, leading to the release of polymorphonuclear leukocytes and protein leakage, which signal inflammation. In addition, carbon nanotubes can form granulomas *in vivo*, leading to toxicity and chronic granulomatous disease, and studies have shown that mice exposed to single-walled carbon nanotubes in the airways develop pulmonary obstruction and granulomas (Czarny et al., 2014).

### 4.3 Pulmonary toxicity

Inhalation of tiny nanoparticles, primarily through the respiratory system, is now considered the primary method of introducing carbon nanotubes into the body (Foldvari and Bagonluri, 2008). Various hypotheses postulate that the unique physical properties of carbon nanotubes may be responsible for their respiratory toxicity in animals (Manke et al., 2014). Important physicochemical aspects, such as particle size, functionalization, and dispersion, greatly influence the pulmonary toxicity profile of carbon nanotubes after inhalation. Wichter et al. (Polimeni et al., 2015) showed that the introduction of single-walled carbon nanotubes (SWCNT) into rat lungs via endotracheal injection resulted in temporary inflammatory and cytotoxic responses. In addition, the use of carboxyl-functionalized SWCNT was associated with increased reactive oxygen species (ROS) production, elevated serum aminotransferase (ALT/AST) and alkaline phosphatase (ALP) activities, and elevated lipid peroxide levels. A series of studies have emphasized that the interaction of SWCNT with epithelial lung cell lines promotes cytotoxic and inflammatory responses (Srivastava et al., 2015).

Taken together, these observations suggest that the toxicity of single-walled nanotubes (SWNTs) may arise primarily from the accumulation of fine nanoparticles within the walls of alveolar and tracheal tissues. The small size of these particles prevents them from settling naturally. Instead, they tend to accumulate in clusters along the inner walls of the airways. This deposition induces the formation of tumors in the airway lining. In some cases, granulomas have been reported in rats (Boyles et al., 2015). This comprehensive story emphasizes the need for thorough evaluation and regulation of respiratory management of carbon nanotubes to reduce potential health hazards.

### 4.4 Hepatotoxicity

**FIGURE 9:**
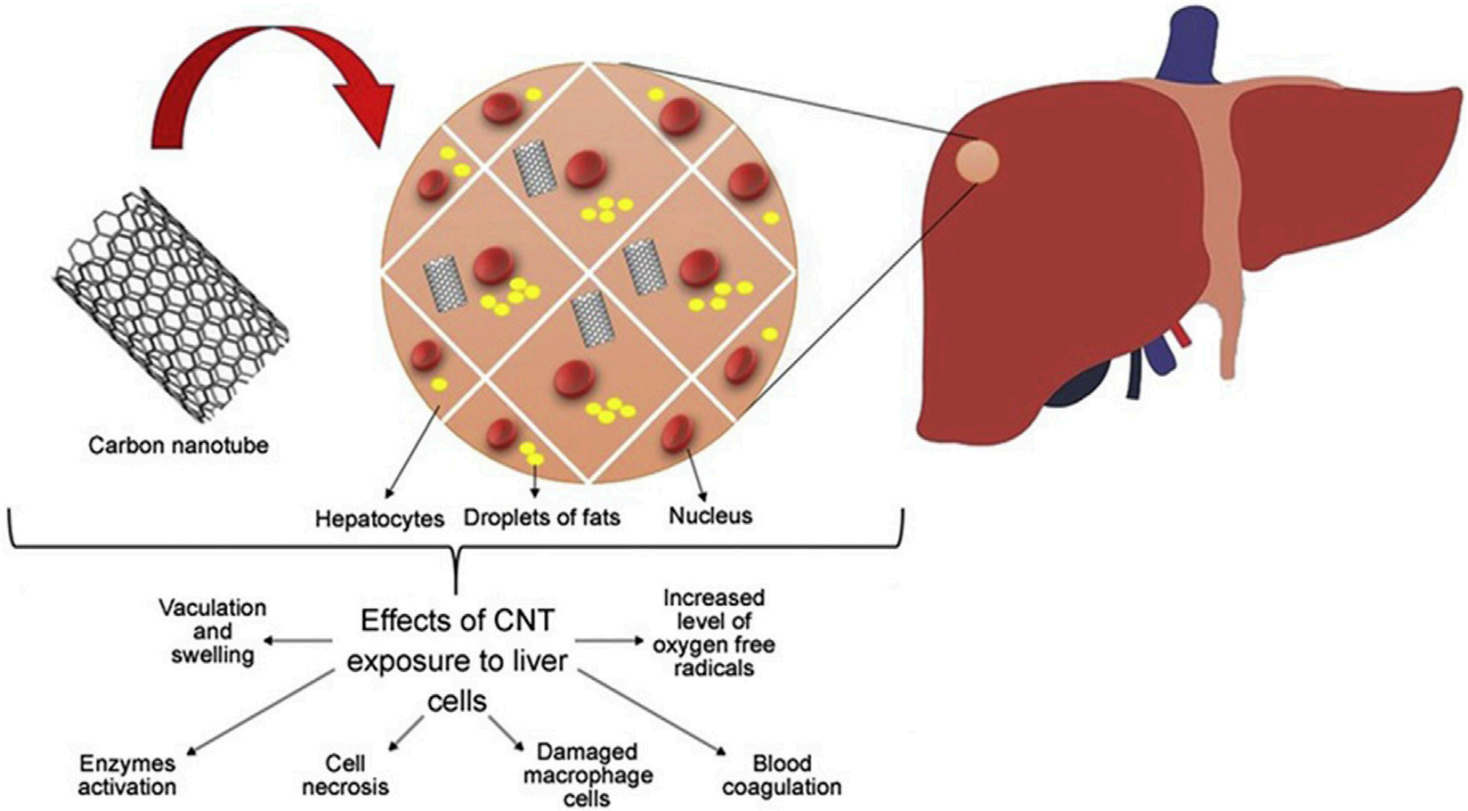
Effect of carbon nanotubes on liver cells.

The liver performs many important functions that are vital to maintaining human health. The liver plays a vital role in regulating blood sugar levels by converting excess glucose into glycogen for storage. In addition, it helps in the metabolism of fats and proteins. Meanwhile, Kupffer cells and other immune cells in the liver are involved in the immune response, helping to clear pathogens and infections (Principi et al., 2016). The liver breaks down and removes toxins and drugs from the body. The functions of the liver include converting toxic substances into harmless or more easily excreted forms. Studies have shown that exposure to carbon nanotubes can lead to liver dysfunction characterized by Kupffer cell activation, blood coagulation, inflammation, and elevated levels of oxygen free radicals (Awasthi et al., 2013). In a mouse model, this exposure prompted the release of pro-inflammatory and inflammatory response substances from Kupffer cells, as shown in Figure 9, suggesting that carbon nanotubes may be a major cause of liver damage.

### 4.5 Central nervous system (CNS) toxicity

The exploration of carbon nanotubes as carriers for drug delivery to the central nervous system (CNS) has been an important area of scientific inquiry. Although carbon nanotubes exhibit low solubility in water in their unaltered form, through careful functionalization and modification they can be engineered to cross the robust blood-brain barrier (BBB), opening up new possibilities for therapeutic interventions within the CNS (Elsori et al., 2023). The unique physical and chemical properties of carbon nanotubes (Du et al., 2017), such as their excellent electrical conductivity, strength, and surface area, have prompted research into their potential diagnostic applications in CNS-related diseases such as Alzheimer’s disease and Parkinson’s disease. These properties offer unique opportunities for advances in the detection and monitoring of such neurological disorders (Cai, 2014). Carbon nanotubes allow for more accurate and earlier detection of biomarkers and disease-induced changes, which may aid in diagnostic and therapeutic strategies.

The exploration of carbon nanotubes for medical applications has not been without drawbacks, particularly about their interaction with the central nervous system. These concerns focus on the potential toxicity of carbon nanotubes, emphasizing the need for thorough research and understanding of their effects on neural tissue and function (Go et al., 2014). Carbon nanotubes can enter the central nervous system (CNS) through a variety of pathways, including crossing the blood-brain barrier and blood-spinal cord barrier via the body circulation. In addition, they can enter the brain through the nasal and olfactory pathways as well as the trigeminal nerve branches in the olfactory and respiratory regions.

**FIGURE 10:**
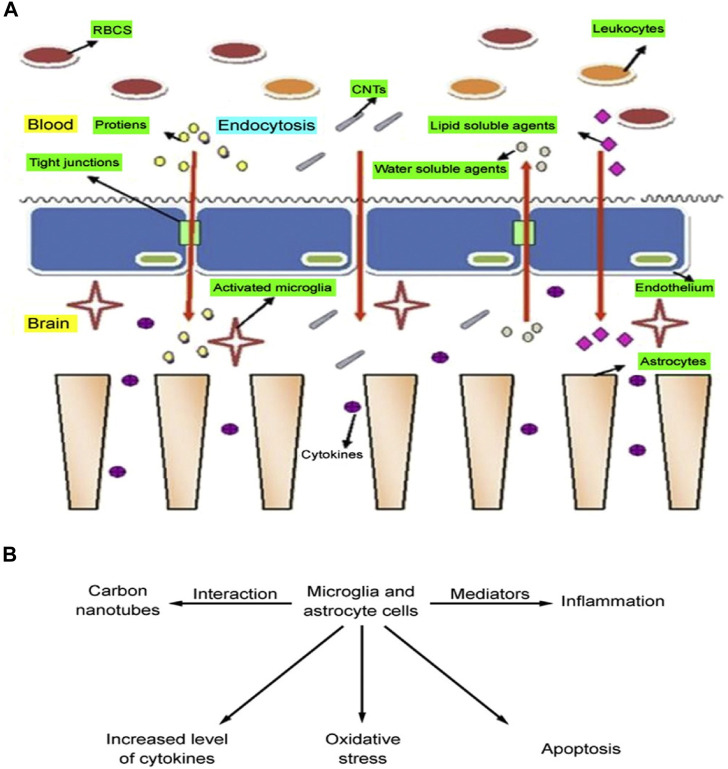
**(A)** Entry pathway of carbon nanotubes across the blood-brain barrier. **(B)** Toxicity from carbon nanotube interactions with brain cells.

Upon interaction with brain cells, carbon nanotubes stimulate microglia and astrocytes to release a range of mediators/chemicals that may lead to inflammation, apoptosis, or oxidative stress in the brain (Bardi et al., 2013). Multi-walled carbon nanotubes (MWCNT) exhibit similar interactions with neural tissues with a toxicity profile comparable to that of single-walled carbon nanotubes (SWCNT). Kafa et al. conducted an in-depth study on the interaction of multi-walled carbon nanotubes with neural tissues. This study involved injecting multi-walled carbon nanotubes directly into the brains of mice to study neuroinflammatory responses under *in vivo* conditions, furthering our understanding of how these nanotubes affect neural health (Zhao et al., 2019). When multi-walled carbon nanotubes (MWCNT) are absorbed, they trigger a range of neuroinflammatory responses. This particular study observed a significant increase in inflammatory cytokines in the cortex following MWCNT injection, as shown in Figure 10. This finding underscores the significant biological effects of MWCNT on neural tissue. There is growing evidence that carbon nanotube surface oxidation may play an important role in the replenishment of energy, focusing on elevated cytokine levels and glial cell activation. inflammatory response within brain cells. This story emphasizes that a thorough examination of the interactions of carbon nanotubes with neural tissues is essential to ensure the safe and effective use of carbon nanotubes in CNS-related diagnostics and therapeutics.

### 4.6 Nephrotoxicity

**FIGURE 11:**
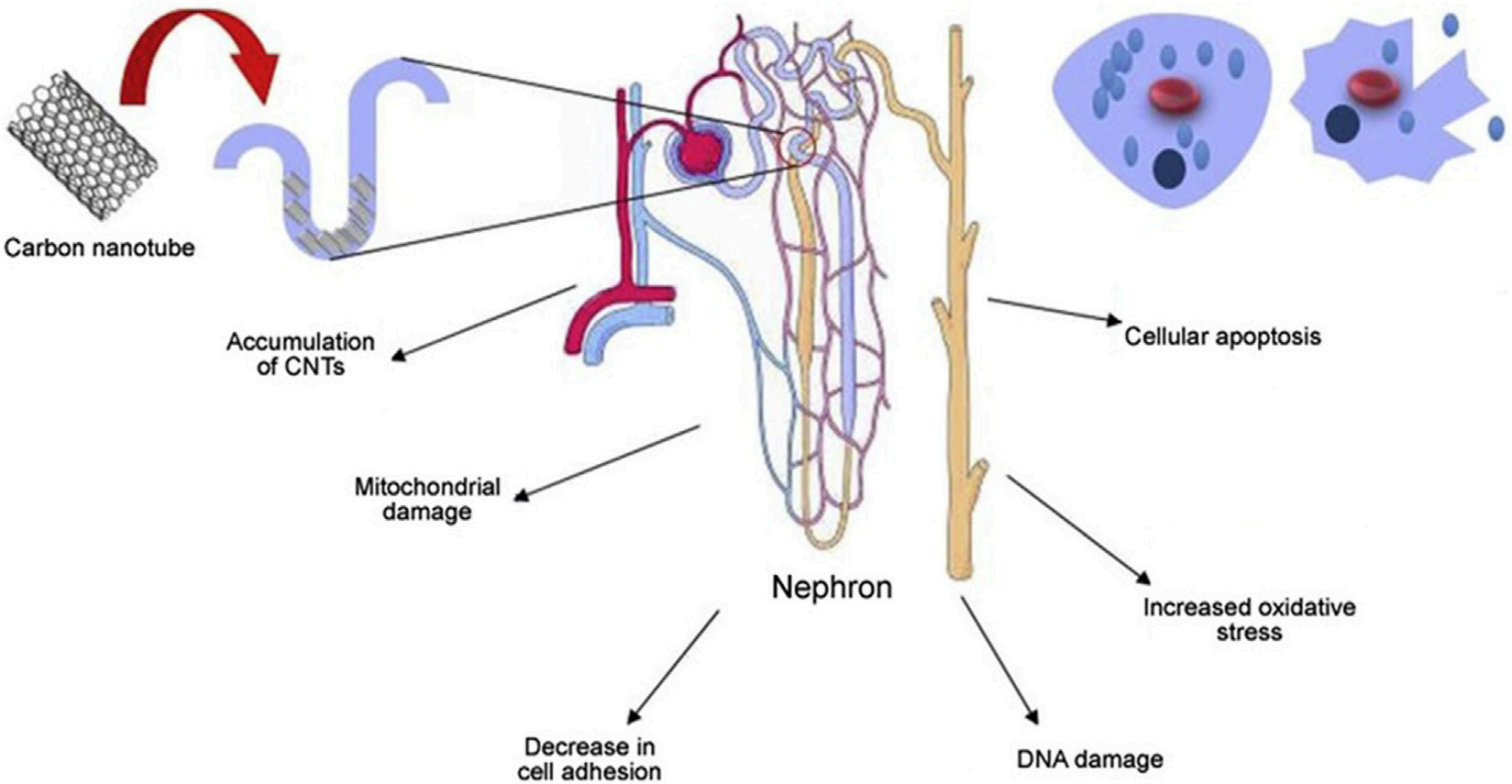
Toxicity studies of carbon nanotubes on the renal system.

The challenge in addressing carbon nanotube toxicity lies primarily in their accumulation in the body. Studies have shown that organs responsible for detoxification, such as the kidneys, are more likely to accumulate these nanotubes. Given the critical role of the kidneys in eliminating toxins, the higher risk of carbon nanotube accumulation in these organs highlights the need for comprehensive research and mitigation strategies to address the potential health effects of carbon nanotubes (van Angelen et al., 2013) and may lead to nephrotoxicity, as shown in Figure 11 In a biocompatibility study of single-walled carbon nanotubes (SWCNT), Cui et al. investigated how single-walled carbon nanotubes interact with human embryonic kidney cells (HEK-293). Their results showed an increase in apoptosis, suggesting that single-walled carbon nanotubes have a potentially detrimental effect on these kidney cells. This study provides valuable insight into the cellular effects and safety of single-walled carbon nanotubes in a biological environment. The treated cells also exhibited reduced cell adhesion. Carbon nanotubes affect the cell cycle, the sequence of phases in which cells replicate and divide. The cycle typically consists of the G1 phase, S phase (where DNA replication occurs), G2 phase, and M phase (actual cell division). Interactions with carbon nanotubes can affect this natural progression, thereby influencing the process of cell development and division. Carbon nanotubes may disrupt the cell cycle by causing uncontrolled expression of the P16 protein. This can lead to the inhibition of key cell cycle proteins such as cell cycle-dependent kinase 2 (CdK2), cell cycle-dependent kinase 4 (CdK4), and cell cycle regulatory proteins (CdKr). As a result, this leads to fewer cells entering the S phase and causes the cell cycle to stall in the G1 phase, which prevents progression through the DNA replication phase. This means that cells are unable to enter the DNA replication phase, which affects cell division and proliferation (Tang et al., 2012). In an experimental study involving cell lines, the application of multi-walled carbon nanotubes (MWCNT) was found to increase the production of IL-6 and IL-8. These cytokines are known for their role in promoting inflammation and immune responses, suggesting that multi-walled carbon nanotubes may trigger inflammatory processes at the cellular level. Multi-walled carbon nanotubes may also contribute to cellular DNA damage by increasing oxidative stress and causing mitochondrial damage. Mitochondria are important organelles responsible for cellular energy production, and damage to them may lead to decreased cellular energy and increased cytotoxicity (Zamani et al., 2021).

### 4.7 Spleen toxicity

The spleen plays a vital role as a secondary lymphoid organ and is part of the reticuloendothelial system (RES), which is integral to the immune response in the blood. The organ is key to regulating immune function and filtering the blood. In their study, the researchers administered water-soluble multi-walled carbon nanotubes (MWCNT) to mice via intravenous injection. The dose was set at 1 mg/kg/day for 2 weeks, and the mouse model was used to explore the effects of multi-walled carbon nanotubes as they are introduced into the body through the bloodstream. Subsequent analysis revealed significant inflammation and immunotoxicity in the spleen, the main immune organ, suggesting that multi-walled carbon nanotubes affect the immune system. The only change from the control group was the movement of nanoparticles from the red marrow region to the white marrow region. This may be because the size and shape of the carbon nanotubes allow them to penetrate the cells and move from the red medulla region to the white medulla region. Carbon nanotubes may be perceived as foreign substances and trigger an immune response. The large number of immune cells such as lymphocytes and macrophages in the white medulla region may interact with the carbon nanotubes, causing them to be transported to the white medulla region and triggering an adaptive immune response (Rai et al., 2021). Although nanoparticles activated the adaptive immune response in the study, they did not significantly affect the phagocytic activity of the reticuloendothelial system. In addition, the levels of certain oxidative stress markers (e.g., glutathione, superoxide dismutase, and malondialdehyde) remained largely unchanged. This suggests a subtle interaction of these nanoparticles with the immune and oxidative stress mechanisms of the human body. Multi-walled carbon nanotubes may affect the immune system, especially causing splenic inflammation and immunotoxicity. Meanwhile, dispersion and chemical treatment of nanoparticles (e.g., functionalization) may affect their toxicity. However, long-term effects may require further studies to be fully understood.

### 4.8 Toxicity summary

Despite the important use and role of single- and multi-walled carbon nanotubes in cancer, detailed studies under a variety of biological conditions have not yet fully elucidated the exact mechanisms of their toxicity. Numerous *in vitro* and *in vivo* studies have been conducted, but the precise causality behind carbon nanotube toxicity remains somewhat elusive. However, it has been hypothesized that the accumulation of carbon nanotube particles and the resulting reactive oxygen species may be the key factors contributing to the observed toxic effects. Given the growing concern over the toxicity of carbon nanotubes, a comprehensive assessment of their interactions with biological systems is essential to ensure their safe and effective use in a variety of applications.

## 5 Conclusion and outlook

Nanotechnology has attracted great attention and interest in the field of medicine and biology, and during the 20 years of its application in biomedicine, progressively modified carbon nanotubes have possessed better biocompatibility and multimodal functionality. They have the advantages of high drug loading, good penetration, photothermal ablation, and inherent diagnostic capabilities. This makes them an excellent vehicle for cancer therapy and opens up opportunities for “customized medicine”, where diagnostic as well as therapeutic recommendations can be made based on the molecular characteristics of the patient. Many studies have demonstrated that CNTs can be used in different cancer imaging such as PA imaging, MRI, Raman imaging, radionuclide imaging, and NIR fluorescence imaging for cancer diagnosis. In addition, when used in combination with other diagnostic reagents, CNTs can be used in nano biosensors for early detection of various types of cancers such as pancreatic, liver, and ovarian cancers with high specificity. The use of carbon nanotubes, which are comparable to the scale of biomolecules, can be an important tool for the targeted delivery of drugs.

Any new tool is like a double-edged sword with two sides; the risk of nanodevices for medical use comes from their potential toxicity, and knowledge of the absorption, distribution, metabolism, and excretion properties of carbon nanotubes in the body is essential. A comprehensive evaluation of the safety of nano-formulations, optimization of drug loading, and reduction of potential toxicity are necessary steps to maximize the therapeutic effect on cancer. For example, free CNTs retained in the body through the bloodstream may cause secondary damage to the cardiovascular, liver, and kidneys. Therefore, we cannot ignore their non-biodegradability and cytotoxicity in clinical applications. Of course, some studies have shown that loading specific proteins on the surface of CNTs stimulates the release of MPO from neutrophils, which leads to the degradation of CNTs and ultimately achieves the attenuation effect. This is also an important future research direction for surface-modified CNTs.

We have thoroughly investigated the potential of carbon nanotubes in the field of cancer therapeutics, their applications, and the limitations they have. Their excellent physical and chemical properties make them a vehicle for cancer therapy. However, the excellent properties of carbon nanotubes are accompanied by unknown risks. Future studies will further explore the functionalization and application of carbon nanotubes to achieve higher therapeutic efficacy and greater safety.
