# Supplementary material for: Nanotechnological advances in cancer: therapy a comprehensive review of carbon nanotube applications
Source: Front Bioeng Biotechnol. 2024 Mar 6;12:1351787. doi: 10.3389/fbioe.2024.1351787 (PMC10984352; doi:10.3389/fbioe.2024.1351787)
Supplement: Supplementary file 1 [file DataSheet1.PDF]

Advances in Carbon Nanotubes in Cancer		
Advantages of carbon nanotubes in cancer applications	Carbon nanotubes in cancer diagnostics	Photoacoustic imaging (PAI), fluorescence imaging (FI), and Raman imaging<br>Modification of carbon nanotubes using biomarkers such as attached antibodies and detection of markers.<br>An innovative approach combining quantum defect functionalized nanotube arrays and machine learning techniques.
	Carbon nanotubes in cancer therapy	Drug delivery, e.g., delivery of doxorubicin (DOX) and polyethylene glycolic SWNTs<br>Targeted therapy using hyperbranched multifunctional carbon nanotube carriers.<br>Modified homogeneous manganese dioxide and photosensitizer color aluminum phthalocyanine (Ce6) for enhanced MRI-guided and TME-responsive phototherapy
Risks of carbon nanotubes in cancer applications	Effects of carbon nanotubes on the organs in the flow of the body	Carbon nanotube deposition in myocardial vascular tissue leads to increased levels of oxidative stress and inflammation.
		Within cells, carbon nanotubes can form granulomas in vivo, leading to toxicity and chronic granulomatous disease.
		In the lungs, the airway lining collects in clusters. This deposition induces the formation of tumors in the lining of the airways.
		In the liver, carbon nanotubes cause Kupffer cell activation, blood clotting, inflammation and elevated levels of oxygen free radicals.
		In the nervous mesosystem, carbon nanotubes lead to a significant increase in inflammatory cytokines in the cortex.
		In the kidney, multi-walled carbon nanotubes may also contribute to cellular DNA damage by increasing oxidative stress and causing mitochondrial damage.
		In the spleen, multi-walled carbon nanotubes may have an effect on the immune system, especially causing splenic inflammation and immunotoxicity.
